# A Real-world Patient Registry for Oritavancin Demonstrates Efficacy and Safety Consistent With the Phase 3 SOLO Program

**DOI:** 10.1093/ofid/ofy051

**Published:** 2018-03-19

**Authors:** Mark Redell, Greg Moeck, Christopher Lucasti, Stephanie Durso, Cynthia Kennedy, Karen Fusaro, Jeff Loutit, Michael Dudley

**Affiliations:** 1 The Medicines Company, Parsippany, New Jersey; 2 The Medicines Company, St. Laurent, Quebec, Canada; 3 South Jersey Infectious Diseases, Somers Point, New Jersey; 4 The Medicines Company, San Diego, California

**Keywords:** ABSSSI, oritavancin, registry, skin infections

## Abstract

**Background:**

Oritavancin is a lipoglycopeptide used in the treatment of acute bacterial skin and skin structure infections (ABSSSIs) in adults. To characterize its use in patients in the postapproval setting, a patient registry was developed.

**Methods:**

Data collected in an ongoing retrospective observational registry are used to evaluate the utilization, outcomes, and adverse events (AEs) associated with oritavancin for the treatment of infections presumed or confirmed to be caused by gram-positive (GP) bacteria in clinical practice.

**Results:**

Data for 112 patients from 8 sites were collected. All patients received a single 1200-mg dose of oritavancin mostly in an infusion center. Infection type included cellulitis (67.0%), cutaneous abscess (21.4%), and wound (4.5%). Most patients (72.3%) received 1 or more antimicrobial agents for the index GP infection within 28 days prior to oritavancin treatment. Of positive cultures obtained prior to oritavancin administration, methicillin-resistant *Staphylococcus aureus* was the predominant pathogen (78.4%). A positive clinical response was observed in 92.8% of patients, and microbial eradication was observed in 90.0% of patients with post-therapy cultures. Within 28 days following oritavancin administration, 4 (3.6%) patients were hospitalized for failure of treatment of the index infection. Five (4.5%) patients experienced 1 or more possible drug-related AEs, which were consistent with types previously reported. There were no drug-related serious AEs reported.

**Conclusions:**

Clinical and microbiologic outcomes and safety of single-dose oritavancin 1200 mg were similar in this older patient population with multiple comorbid conditions to those observed in the phase 3 SOLO trials.

Oritavancin (Orbactiv; The Medicines Company, Parsippany, NJ) is a bactericidal lipoglycopeptide antibiotic that is approved in the United States and European Union for the treatment of adult patients with acute bacterial skin and skin structure infection (ABSSSI) caused by designated gram-positive pathogens including methicillin-resistant *Staphylococcus aureus* (MRSA). Two identical phase 3, international, randomized, and double-blind trials (SOLO I and SOLO II) demonstrated that a single 1200-mg intravenous (IV) dose of oritavancin was noninferior to vancomycin at a dose of 1 g or 15 mg/kg every 12 hours for 7 to 10 days for the treatment of ABSSSI [1–4]. Oritavancin was introduced in the US market in late 2014.

A patient registry for oritavancin (Clinical and Historic Registry and Orbactiv Medical Evaluation [CHROME]) was established to characterize the use of oritavancin in postmarketing real-world settings. CHROME is a multicenter, multiyear, retrospective observational study to characterize the population of adult patients who have received oritavancin for the treatment of infections due to presumed or confirmed gram-positive bacteria and to describe the associated clinical and microbiologic outcomes and safety. We present results from the first phase of the CHROME registry.

## METHODS

### Study Design and Patient Population

Patients who received at least 1 dose of oritavancin were eligible for enrollment. Each site enrolled at least 10 consecutive patients who had received at least 1 dose of oritavancin between October 2014 and April 2016. Sites collected patient baseline demographics, vital signs at the time of oritavancin infusion, baseline laboratory data, infection onset dates and infection classification, extent of infection area (direct measurement or comparative estimation), infection management procedures, pathogens, pre- and post-therapy antibiotics, clinical and microbiologic outcomes, date and time of oritavancin administration (including infusion start and stop times), patient disposition following oritavancin administration, and drug-related adverse events. Patients were enrolled regardless of site of care, which included infusion centers, clinics, emergency departments, and observation and inpatient hospital beds.

### Inclusion and Exclusion Criteria

To be enrolled in CHROME, patients had to (1) be treated with oritavancin for a suspected or confirmed gram-positive infection as monotherapy or part of a broader regimen and (2) have received the last dose of oritavancin at least 60 days prior to data entry into the electronic case report form (eCRF). Waivers of informed consent were obtained from Institutional Review Boards overseeing participating sites, given the retrospective nature of the study and de-identification of patient information collected through the data entry process and final aggregation of data.

### Safety Assessments and Reporting

Safety definitions were established by a Global Pharmacovigilance committee a priori and reflected those established for the phase 3 SOLO studies in adults with ABSSSI. Safety data were collected up to 60 days following the last dose of oritavancin. Adverse events with a reasonable possibility of a causal relationship to oritavancin, as assessed by the investigator, were reported and categorized based on their seriousness and severity according to definitions outlined in the protocol. Serious adverse events (SAEs) were defined as events that resulted in death, were life-threatening, resulted in persistent or significant disability or incapacity, required prolonged hospitalization, or were medically significant events that may have jeopardized the patient and may have required medical or surgical intervention to prevent 1 of the previously listed outcomes. SAEs, and seriousness and severity of adverse events (AEs), were collected for regulatory reporting. All SAEs, adverse events of special interest (AESIs), and pregnancies within 60 days of oritavancin infusion were reported by the investigator within 24 hours of discovery.

### Data Collection Form and Process

Investigators were trained on the use of a standardized electronic data capture instrument, which served to build case report forms. Sites utilized eClinicalOS (IBM Clinical Development; Durham, NC) as the data entry platform. All data were entered remotely by study personnel at the investigator’s site. Site audits were conducted remotely through a series of validation steps and data queries.

### Clinical and Microbiologic Assessments

The protocol provided a 28-day window to capture clinical and microbiologic outcomes. Clinical categories of efficacy assessed between end of infusion and 28 days following the dose of oritavancin were defined as one of the following: clinical cure (clinical signs and symptoms resolved), clinical improvement (partial resolution of clinical signs and symptoms), clinical failure (inadequate resolution or new or worsening clinical signs and symptoms, including need for additional antibiotic therapy for treatment of the baseline infection), or nonevaluable (unable to determine response because the medical record did not contain the necessary information to determine cure, improvement, or failure).

Microbiological response categories for assessments conducted between the end of infusion and 28 days following the dose of oritavancin considered only gram-positive pathogens believed to be related to the infection process. Microbiological response was defined as either microbiologic eradication (documentation of a negative bacterial culture from the same site as the initial positive baseline culture) or microbiologic persistence (bacterial growth of the same organism from the same site as the initial positive baseline culture). Patients with baseline pathogens were not required to have post-therapy cultures in order to evaluate eradication or persistence, and these were performed as clinically indicated.

### Statistics

Results were descriptive, and no statistical analysis was performed on the data presented herein or in comparisons with the SOLO pooled data. Although not described a priori in the analysis plan, comparison with selected data from the pooled SOLO trials is presented alongside CHROME in the data tables.

## RESULTS

### Patient Population and Demographics/Baseline Characteristics

Data for 112 patients were collected from 8 health care sites. Though no restrictions were placed on type of infection treated with oritavancin, all 112 cases entered by sites were for skin and soft tissue infections. All patients received a single 1200-mg dose of oritavancin, with 86.6% (97/112) of patients treated in a hospital-owned or physician-owned infusion center. An additional 12 patients were treated with oritavancin in the emergency department (10.7%).

Demographics and baseline patient characteristics are presented in Table 1. Comparative data from the pooled SOLO program are provided when specific categories could be matched and data were available.

**Table 1.** Demographics and Baseline Characteristics for Oritavancin-Treated Patients in the CHROME Registry and SOLO Clinical Program

Characteristic^a^	CHROME (n = 112 patients)	Pooled SOLO^b^ (n = 978)
Age, y		
Mean (SD)	58.6 (17.0)	45.6 (13.8)
Median	60.0	46.0
Range	18 – 96	18 – 89
No. (%) age ≥65 y	44 (39.3)	86 (8.8)
Sex, %		
Male	53.6	65.3
Female	46.4	34.7
Race (n = 108), %		
White	91.7	64.4
Body weight (n = 111), kg		
Mean (SD)	98.4 (29.4)	79.0 (22.7)
Median	97.5	75
Range	48 – 223	35 – 200
Body mass index (n = 109), kg/m^2^		
Mean (SD)	33.0 (9.6)	27.7 (7.6)
Median	31.6	26.2
Range	16–65	15–74
Meets SIRS criteria, No. (%)^c^	4/112 (3.6)	169 (17.3)
Temperature ≥38^o^C	0/104 (0)	186/978 (19.0)
WBC count >12 000 mm^3^	11/52 (21.2)	216/887 (24.4)
Common infection management procedures, % (n/N)	29 (33/112)	14.1 (138/978)
Incision and drainage of abscess	63.6 (21/33)	58.0 (80/138)
Deep tissue surgical debridement	21.2 (7/33)	20.3 (28/138)^d^
Superficial surgical debridement	15.2 (5/33)	
Hospitalized, % (n/N)	Prior to receipt of oritavancin: 10.7 (12/112)^e^	Study postrandomization: 60 (586/978)^e^
Patients receiving antibiotics prior to oritavancin, % (n/N)	70.5 (79/112)^f^	19.6 (192/978)^f^
Concomitant medical conditions, %		
Vascular disorders	55.4	
Hypertension	44.6	
Diabetes	37.5	14.2
Intravenous drug use	ND	29.2
Hyperlipidemia	25.1	
Neoplastic disease	17.9	
Microbiology		
Baseline infection site culture recovery rate,^g^ % (n/N)	64.8 (46/71)	63.4 (620/978)
Confirmed gram-positive pathogen at baseline,^h^ % (n/N)	96.0 (48/50^i^)	53.3 (529/992 [ITT])
*Staphylococcus aureus*	77.1 (37/48)	89.2 (472/529)
MRSA, % (n/N)	78.4 (29/37)	38.6 (204/529)
MSSA, % (n/N)	21.6 (8/37)	50.7 (268/529)
Infection type, No. (%)		
Cellulitis	67.0 (75)	39.6 (387)
Cutaneous abscess	21.4 (24)	31.5 (288)
Wound infection	4.5 (5)	28.9 (283)
Other^j^	7.1 (8)	NA

Abbreviations: CHROME, Clinical and Historic Registry and Orbactiv Medical Evaluation; MRSA, methicillin-resistant *Staphylococcus aureus*; MSSA, methicillin-sensitive *Staphylococcus aureus*; SIRS, systemic inflammatory response syndrome; WBC, white blood cell.

^a^Characteristic applies to 112 patients unless otherwise stated.

^b^Reference 3.

^c^Systemic inflammatory response syndrome is defined as 2 of the following: temperature >38°C, pulse >90 beats per minute, respiratory rate >20 breaths per minute, white blood cell count >12 000 mm^3^ or <4000 mm^3^, or >10% bandemia.

^d^For SOLO patients, the infection management procedure most closely related to deep tissue surgical debridement was major surgical debridement under general anesthesia.

^e^For CHROME, hospitalization refers to the care necessary to treat the lesion within 28 days prior to oritavancin administration; for the pooled SOLO population, hospitalization refers to those who received some of their postrandomization study drug treatment in an inpatient setting; this does not infer that these patients were hospitalized prior to receipt of oritavancin.

^f^CHROME, within 28 days prior to oritavancin; SOLO, within 14 days prior to screening.

^g^Seventy-one patients with infection sites cultured.

^h^Of all pathogens identified.

^i^Fifty total pathogens recovered from 46 cultured sites; 48 were gram-positive.

^j^Other infection type included chronic osteomyelitis (2), bursitis (1), tenosynovitis (1), diabetic foot (1), bacterial arthritis (1), lymphadenitis (1), and nonspecific impaired healing (1).

Comparing CHROME and SOLO populations, more patients in CHROME than in the SOLO pool were age 65 years and older (39.3 vs 8.8%, respectively), were white (91.7 vs 64.4%, respectively), were obese (20% greater body mass index in CHROME), were hospitalized within 28 days prior to receipt of oritavancin (70.5 vs 19.6%, respectively), were diabetic (37.5 vs 14.2%, respectively), and had cellulitis (67.0 vs 39.6%, respectively). In contrast, fewer patients in CHROME than in the SOLO pool met systemic inflammatory response syndrome criteria (3.6 vs 17.3%, respectively), were febrile at baseline (0.0 vs 19.0%, respectively), and had wound infection (4.5 vs 28.9%, respectively). While the incidence rates of leukocytosis were similar, no patient in CHROME presented with fever (temperature ≥ 38^o^C). The most common underlying medical conditions in patients were general vascular disorders, hypertension, diabetes, hyperlipidemia, and neoplastic disease, present in 55.4%, 44.6%, 37.5%, 25.1%, and 17.9% of patients, respectively.

There were 123 infections in 112 patients (data not shown). Several patients presented with infections at more than 1 anatomic location. The majority of infections were diagnosed in a physician’s office (49.5%) or emergency department (38.7%). Infections of the extremities (ie, arm, hand, leg, or foot) were the most common locations observed (78.0%) compared with nonextremity sites (ie, chest, trunk, back, buttocks), which were far less common (8.9%). Lower extremity infections predominated (59.3%).

Infections that could not be classified within the 3 major infection types in CHROME are provided in Table 1. Six patients in CHROME received a single 1200-mg dose of oritavancin to manage primary skin infections that involved deep tissues, including orthopedic infections, infected amputation surfaces, and infected areas contiguous to skin grafts. Two patients received a single dose of oritavancin as follow-on therapy for presumed gram-positive complicated skin and soft tissue infections, including 1 patient with a left-ventricular assist device exit wound infection requiring surgical debridement prior to removal and a second patient who had surgical removal of an infected right shoulder implant.

### Microbiology

Table 1 describes the microbiology in CHROME patients. Of the 112 patients enrolled, 71 (63.4%) had infection sites cultured. Forty-six patients (41.1%) had positive cultures for at least 1 bacterial pathogen; gram-positive bacteria were recovered in 37 patients, 7 patients had a mixed gram-positive/gram-negative infection, and 2 patients had a gram-negative infection only. In total, 50 bacterial isolates were recovered—48 isolates were gram-positive and 2 gram-negative. Of 37 patients with *Staphylococcus aureus*, 78.4% (29/37) had MRSA. Two patients were identified with infections due only to gram-negative bacilli, but gram-positive cocci were suspected due to the nature of the infection. Baseline bacteremia due to methicillin-sensitive *Staphylococcus aureus* (MSSA) was observed in 1 of 25 patients who had blood cultures collected. The dominance of MRSA in CHROME patients contrasts sharply with SOLO, in which the latter study showed that of patients with positive cultures for *S. aureus*, 38.6% had MRSA.

### Use of Other Antimicrobial Agents

Exposure to other antibiotics was common in CHROME-enrolled patients, while this was much less common in SOLO (19.6% of patients). Most patients (70.5%) received at least 1 systemic antimicrobial agent for a skin and skin structure infection within 28 days prior to treatment with oritavancin. Cephalosporins (58.0%), vancomycin (46.9%), trimethoprim-sulfamethoxazole (25.9%), clindamycin (21.0%), and penicillins (16.0%) were the most common agents prescribed. Ceftaroline accounted for 19.1% of cephalosporins used prior to the first dose of oritavancin. Clinical and/or microbiologic failure (55.8%) and completion of therapy against the offending gram-positive infection (19.0%) were the most commonly stated reasons for discontinuing prior antibiotics in favor of single-dose administration of oritavancin. Eight patients (10.4%) were switched to oritavancin as a result of adverse events or intolerance to prior antibiotics. The number of nonoritavancin antibiotic regimens were not collected, and clinical outcomes were not correlated with specific courses of therapy.

### Clinical and Microbiologic Outcomes

Data on clinical response were available for 111 patients (Table 2). Clinical response was noted for 103/111 (92.8%) evaluable patients. Among 8 patients classified as clinical failures, all received additional antimicrobial therapy within the 28-day period following receipt of oritavancin.

**Table 2.** Clinical and Microbiologic Outcomes for Oritavancin-Treated Patients in the CHROME Registry and SOLO Clinical Program

Outcome	CHROME (n = 112), % (n/N)	Pooled SOLO (n = 978), % (n/N)
Clinical success^a^	92.8 (103/111)	92.6 (760/821)^b^
Clinical failure	7.2 (8/111)	7.4 (61/821)
Post-therapy microbiologic assessment in 30 patients^c^		Not available; clinical response by pathogen at defined end points
Microbiologic eradication	90.0 (27/30)	
Microbiologic persistence	10.0 (3/30)	

Abbreviation: CHROME, Clinical and Historic Registry and Orbactiv Medical Evaluation.

^a^Clinical success in the SOLO study (CE population at post-therapy follow-up) was defined as investigator-assessed clinical success at post-therapy evaluation at days 14 to 24 (7 to 14 days from end of blinded therapy). A patient was categorized as a clinical success if the patient experienced a complete or nearly complete resolution of baseline signs and symptoms related to primary ABSSSI site such that no further treatment with antibiotics was needed. CHROME definitions excluded the need for additional antibiotics. Clinical success includes clinical cure and clinical improvement, as assessed within 28 days following oritavancin administration.

^b^Reference 3. Clinically evaluable population at post-therapy follow-up as defined above.

^c^In CHROME, microbiologic assessment includes laboratory-confirmed microbial eradication of the same baseline pathogen at the site of the initial infection or microbiologically confirmed persistence.

Within 28 days following oritavancin administration, 4 (3.6%) patients were hospitalized for worsening or recurrence of the index infection. A fifth patient was seen in the emergency room for further treatment of the index infection. The remaining 3 clinical failures were managed in the ambulatory setting.

A single patient had a bloodstream infection due to MSSA prior to receipt of oritavancin. The bacteremia was felt to be associated with the initial skin infection, and presumptive microbial eradication was attributable to receipt of prior antibiotics. Additional blood cultures were not obtained to confirm eradication. Oritavancin was administered to complete a treatment course.

In 30 patients with follow-up cultures, microbiologic eradication was documented in 27 patients and microbiologic persistence was documented in 3 patients with various complicated skin and soft tissue infections.

### Safety Outcomes

The evaluable safety cohort consisted of 112 patients who received a single dose of oritavancin (Table 3). There were no SAEs reported. Five (4.5%) patients experienced at least 1 drug-related treatment-emergent adverse event (TEAE) considered by the investigator to be definitely related or possibly related to oritavancin. Two of these 5 patients experienced mild hypersensitivity reactions during infusion. Neither patient reported prior exposure to glycopeptides. Infusions were briefly interrupted, patients were provided with supportive care, and the remainder of the infusate was delivered without further incident. Both patients completed the full 1200-mg dose. Relative to the 2 hypersensitivity reactions, 1 patient reported a history of multiple drug allergies. The event occurred 4 minutes into the infusion, with the patient complaining of pounding heart, chest pain radiating to the back, headache, and blurry vision. The infusion was stopped for 15 minutes, and the patient was administered 50-mg diphenhydramine plus 1-g acetaminophen. The oritavancin infusion was resumed at a slower rate, gradually increasing the infusion to the original rate. The oritavancin infusion was completed 4 hours after initiation without further events. The second patient reported no known drug allergies. The hypersensitivity adverse event occurred 2 hours after initiation of the infusion, with the patient complaining of chills, shivering, and shaking. Ondansetron 4-mg IV was administered. The infusion was stopped for 30 minutes before restarting at half the infusion rate. The total time from initiation to completion of oritavancin was 5.5 hours. No additional events occurred after restarting oritavancin.

**Table 3.** Treatment-Emergent Adverse Events for Oritavancin-Treated Patients in the CHROME^a^ Registry and SOLO Clinical Program

Adverse Event	CHROME (n = 112), % (n/N)	Pooled SOLO (n = 976), % (n/N)
Patients with a drug-related adverse event	4.5 (5/112)	27.2 (417/1535)^b^
Patients with a drug-related serious adverse event	0	0.33 (5/1535)^b^
Discontinuation due to any adverse event	0	3.7 (36/976)^c^
Incidence of selected adverse event		
Hypersensitivity	1.8 (2/112)	7.7 (75/976)^d^
Diarrhea	1.8 (2/112)	3.7 (36/976)^c^
Vomiting	0.9 (1/112)	4.6 (45/976)^c^
*Clostridium difficile*–associated diarrhea	0.9 (1/112)	0^c^

Abbreviation: CHROME, Clinical and Historic Registry and Orbactiv Medical Evaluation.

^a^In CHROME, adverse events (AEs) with a reasonable possibility of a causal relationship to oritavancin, as assessed by the Investigator, were reported. In SOLO, AEs for which there was reasonable evidence to suggest a causal relationship between the AE and the study medication and AEs that were considered to be related to the study medication with a high degree of certainty were considered “related.”

^b^Reference 15. Denominator represents total number of treatment-emergent adverse events in 976 patients.

^c^Reference 4.

^d^Data on file.

Three additional patients experienced gastro-intestinal adverse events. One patient was recorded as having diarrhea, and another patient experienced diarrhea and vomiting. The third patient was diagnosed with *Clostridium difficile*–associated diarrhea (CDAD). The patient had a history of recent treatment with multiple oral and injectable antibiotics (levofloxacin, ceftriaxone, clindamycin, and cephalexin) prior to infusion of oritavancin. Thirteen days following the single dose of oritavancin, the patient complained of mild diarrhea, headache, and body aches upon wakening. A polymerase chain reaction test subsequently confirmed *C. difficile*. A 7-day course of metronidazole oral (500-mg TID) was prescribed at the clinic. Diarrhea resolved later the same day as the clinic visit, and the course of metronidazole was completed.

## DISCUSSION

The CHROME registry provides valuable insight into the early clinical use of oritavancin and represents the first analysis of data in patients studied under real-world conditions at multiple health care sites. Comparisons between CHROME and SOLO patient demographics and baseline characteristics, safety, and clinical and microbiologic end points provide assurance that outcomes are similar despite inherent differences in design and patient populations. Future phases of CHROME will continue to provide experiential safety and efficacy data on oritavancin.

Patient comorbidities may predispose to perfusion disorders, leading to compromised antibiotic delivery to infection sites, resulting in lower clinical and microbiologic response rates to treatment [5]. In CHROME, a high number of patients presented with vascular disorders (55%), diabetes (38%), and extreme obesity (30%), advanced age (≥65 years), and neoplastic disease (18%). These comorbid conditions may result in decreased delivery of antibiotics to infection sites, lower host response to infection, and predispose to clinical failure. In SOLO, the incidence rates of advanced age, diabetes, and obesity were considerably lower. Despite these predispositions, clinical success was observed for 93% of patients in CHROME. It is important to note, however, that one-third of patients enrolled in SOLO were identified as intravenous drug users, while this demographic was not captured in CHROME.

The setting of care is determined by many factors, including severity of disease and patient comorbid conditions. Most patients in CHROME received oritavancin in an outpatient infusion care setting, and 71% received systemic antibiotics within 28 days prior to oritavancin. The choice to infuse oritavancin in nonhospital settings may provide patient convenience through consolidation of the remaining antibiotics into a final course using a single long-acting agent. The real-world propensity for oritavancin to be administered to patients with acute bacterial skin and skin structure infection in the outpatient setting reflects a unique option for many patient populations.

The practice of culturing infected skin and soft tissues has not been well studied in real-world settings. Patients in CHROME were frequently cultured (63%, 71 cultures among 112 patients) despite most infections presenting as cellulitis (67.0%), which is not generally considered amenable to culturing unless the lesion is purulent and accompanied by the presence of tissue or exudate at the leading edges [5]. Jenkins et al. [6] implemented a clinical practice guideline for cellulitis and cutaneous abscess, which led to a significant decrease in the requisition of microbiological cultures (80% at baseline vs 66% with intervention, *P* = .003).

While *S. aureus* predominated in both SOLO (89%) and CHROME (77%), the high rate of MRSA in cultures obtained during the CHROME registry, constituting 78.4% of identified *S. aureus* isolates, was not expected. This is especially remarkable given the predominance of cellulitis as the major lesion type in CHROME. The similar culture-positive recovery rates (CHROME, 64.8%; SOLO, 63.4%) suggest that MRSA was a common infecting pathogen in patients with cellulitis in CHROME. While the prevalence of MRSA decreased between 2011 and 2014 among 2 major hospital-acquired infections (central line-associated bloodstream infection [CLABSI] and surgical site infection [SSI]; https://gis.cdc.gov/grasp/PSA/MapView.html [7]), MRSA remains a frequent and concerning pathogen in many patient populations [8]. The predominance of this pathogen in CHROME-enrolled patients reinforces the ongoing need for anti-MRSA agents that enable patients with skin and skin structure infections to be cost-effectively managed as outpatients, without concern for poor medication compliance [9–11].

Most patients in CHROME received prior antibiotic therapy for their infections. Initial therapy with antibiotics other than oritavancin was prevalent (71%), with prior clinical and/or microbiologic failure, completion of a course of therapy, and intolerance or allergy to the initial nonoritavancin antibiotics accounting for almost 85% of those patients transitioned to a single dose of oritavancin. Antibiotic pre-exposure is not generally permitted in clinical trials or is restricted to short half-life agents and limited time frames. Concerning, however, is the significant clinical and/or microbiologic failure rates (55%) that preceded a switch to oritavancin.

Recent studies suggest that oritavancin, when used as a single dose for ABSSSI in the initial treatment of infections suspected to be caused by susceptible gram-positive pathogens, may provide opportunities to lower the health care cost burden in certain scenarios. Anastasio et al. [10] found that oritavancin’s clinical effectiveness was consistent across ABSSSI patient subgroups, that it was consistently associated with lower costs and reduced resource utilization, and that it was associated with a shorter duration of treatment. A cost-minimization model indicated that use of oritavancin in the emergency department or observation setting was associated with substantial cost savings compared with inpatient treatment with vancomycin [12]. Similar results were observed in a decision-analytical model [13]. The comparable efficacy and safety of oritavancin in the ambulatory setting with that observed for patients treated in the inpatient setting [9] suggests that a single 1200-mg dose of oritavancin for the treatment of skin and skin structure infection could likely replace vancomycin and beta-lactams as primary therapy in certain settings.

Clinical success rates achieved in patients in CHROME and SOLO were identical (93%), although time points for this assessment varied—the clinically evaluable population at the post-therapy visit at day 14 to 24 [1–3] in SOLO vs the 28-day window for evaluation in CHROME. Microbiological eradication of the baseline gram-positive pathogen in CHROME was observed in 90% of patients with post-therapy cultures.

Because clinical trials are performed under prospectively defined safety plans and data collection methods [14], adverse reaction rates observed in SOLO cannot be directly compared with rates observed in real-world clinical practice. The SOLO studies reported an overall frequency of TEAEs for oritavancin of 55.3% [4]. Fewer TEAEs were judged by SOLO investigators to be related to oritavancin (27.2%), and drug-related serious adverse events were also low (0.33%) [15]. CHROME investigators were instructed to report adverse events with a reasonable possibility of a causal relationship to oritavancin, resulting in a lower adverse reaction rate in CHROME (4.5%).

A review of the safety population in SOLO revealed hypersensitivity reported in 7.7% (75/976) of oritavancin-treated patients (data on file). Three patients in CHROME experienced either mild hypersensitivity (2 patients) or mild CDAD (1 patient), but none of these events was categorized as serious. While data capture methods and safety assessments differ between clinical trials and registries, the finding of 2 cases of nonserious hypersensitivity in CHROME’s 112 patient population is promising. In addition, no serious drug-related adverse events, including serious hypersensitivity events, were recorded in CHROME among 112 patients treated with oritavancin. Finally, all 112 patients completed the full 1200-mg oritavancin dose, and only 2 required brief interruptions of infusion.

Some of the limitations of a registry such as CHROME include the retrospective, noncomparative, unblinded, and nonrandomized nature of the data from a limited number of sites. Assessment of efficacy was based on a subjective assessment extracted from the medical record by the investigators. Missing data may have been encountered given the 28-day clinical assessment and 60-day safety evaluation windows in an agent administered once, as observed in this first phase of CHROME. Nevertheless, the retrospective nature of the CHROME registry allowed rapid collection of real-world data, where only clinical trial and post hoc analyses are available for oritavancin.

Postmarketing registries of the real-world use of antibiotics provide an additional data source for clinicians to evaluate safety and efficacy in patient populations not studied during clinical development. Future data derived from CHROME will add to the current body of evidence for the clinical and microbiologic efficacy and the safety and tolerability of oritavancin as well as gather additional information associated with the real-world experience of oritavancin used in a variety of settings and infections [16–18].

## CONCLUSION

Treatment with oritavancin in 112 patients in a real-world registry enrolling patients with more risk factors for negative outcomes revealed clinical outcomes identical to the larger phase 3 SOLO program. The microbiological eradication rate was high in a population with MRSA as the predominant pathogen. The experience in CHROME does not signal any safety and tolerability concerns. Cognizant of the differences between clinical trials and real-world registries, the data collected in CHROME appear to establish the safe and effective use of a single dose of oritavancin in the treatment of skin and soft tissue infections.

## References

[1] Corey GR, Kabler H, Mehra P, et al SOLO I Investigators Single-dose oritavancin in the treatment of acute bacterial skin infections. N Engl J Med 2014; 370:2180–90. 24897083 10.1056/NEJMoa1310422

[2] Corey GR, Good S, Jiang H, et al SOLO II Investigators Single-dose oritavancin versus 7-10 days of vancomycin in the treatment of gram-positive acute bacterial skin and skin structure infections: the SOLO II noninferiority study. Clin Infect Dis 2015; 60:254–62. 25294250 10.1093/cid/ciu778

[3] Corey GR, Arhin FF, Wikler MA, et al SOLO I, SOLO II Investigators Pooled analysis of single-dose oritavancin in the treatment of acute bacterial skin and skin-structure infections caused by gram-positive pathogens, including a large patient subset with methicillin-resistant *Staphylococcus aureus*. Int J Antimicrob Agents 2016; 48:528–34. 27665522 10.1016/j.ijantimicag.2016.07.019

[4] The Medicines Company. Prescribing Information – ORBACTIV (Oritavancin) for Injection. Parsippany, NJ: The Medicines Company; 2016.

[5] Stevens DL, Bisno AL, Chambers HF, et al Infectious Diseases Society of America Practice guidelines for the diagnosis and management of skin and soft tissue infections: 2014 update by the Infectious Diseases Society of America. Clin Infect Dis 2014; 59:e10–52. 24973422 10.1093/cid/ciu444

[6] Jenkins TC, Knepper BC, Sabel AL, et al Decreased antibiotic utilization after implementation of a guideline for inpatient cellulitis and cutaneous abscess. Arch Intern Med 2011; 171:1072–9. 21357799 10.1001/archinternmed.2011.29

[7] Centers for Disease Control and Prevention. Antibiotic resistance patient safety atlas. Available at: https://gis.cdc.gov/grasp/PSA/MapView.html. Accessed April 17, 2017.

[8] Rolston KVI Infections in cancer patients with solid tumors: a review. Infect Dis Ther 2017;6:69–83. 28160269 10.1007/s40121-017-0146-1 PMC5336421

[9] Lodise TP, Redell M, Armstrong SO, Sulham KA, Corey GR Efficacy and safety of oritavancin relative to vancomycin for patients with acute bacterial skin and skin structure infections (ABSSSI) in the outpatient setting: results from the SOLO clinical trials. Open Forum Infect Dis 2017;4:ofw274. 28480266 10.1093/ofid/ofw274 PMC5414048

[10] Anastasio PJ, Wolthoff P, Galli A, Fan W Single-dose oritavancin compared to standard of care IV antibiotics for acute bacterial skin and skin structure infection in the outpatient setting: a retrospective real-world study. Infect Dis Ther 2017; 6:115–28. 28078655 10.1007/s40121-016-0145-7 PMC5336420

[11] Eells SJ, Nguyen M, Jung J, et al Relationship between adherence to oral antibiotics and postdischarge clinical outcomes among patients hospitalized with *Staphylococcus aureus* skin infections. Antimicrob Agents Chemother 2016; 60:2941–8. 26926634 10.1128/AAC.02626-15 PMC4862485

[12] Lodise TP, Fan W, Sulham KA Economic impact of oritavancin for the treatment of acute bacterial skin and skin structure infections in the emergency department or observation setting: cost savings associated with avoidable hospitalizations. Clin Ther 2016; 38:136–48. 26708118 10.1016/j.clinthera.2015.11.014

[13] Jensen IS, Lodise TP, Fan W, et al Use of oritavancin in acute bacterial skin and skin structure infections patients receiving intravenous antibiotics: a US hospital budget impact analysis. Clin Drug Investig 2016; 36:157–68. 10.1007/s40261-015-0365-8 PMC4740574 26692006

[14] US Food and Drug Administration. *Guidance for Industry*. Acute Bacterial Skin and Skin Structure Infections: Developing Drugs for Treatment. Silver Spring, MD: FDA; October 2013.

[15] Corey GR, O’Riordan W, Huang NY, et al Time to onset and duration of adverse events in patients with acute bacterial skin and skin structure infection treated with oritavancin – the SOLO studies. Poster 685. Presented at: ID Week 2014; Oct 8–12, 2014; Philadelphia, PA.

[16] Antony SJ, Cooper LG Use of oritavancin (novel new lipoglycopeptide) in the treatment of prosthetic joint infections (PJI): a possible alternative novel approach to a difficult problem. Infect Disord Drug Targets 2017; 17:77–80. 27829328 10.2174/1871526517666161108130148

[17] Johnson JA, Feeney ER, Kubiak DW, Corey GR Prolonged use of oritavancin for vancomycin-resistant *Enterococcus faecium* prosthetic valve endocarditis. Open Forum Infect Dis 2015;2:ofv156. 26677455 10.1093/ofid/ofv156 PMC4677157

[18] Stewart CL, Turner MS, Frens JJ, et al Real-world experience with oritavancin therapy in invasive gram-positive infections. Infect Dis Ther 2017; 6:277–89. 28386776 10.1007/s40121-017-0156-z PMC5446369

